# Development and Characterization of Syngeneic Orthotopic Transplant Models of Obesity-Responsive Triple-Negative Breast Cancer in C57BL/6J Mice

**DOI:** 10.3390/cancers16162803

**Published:** 2024-08-09

**Authors:** Meredith S. Carson, Patrick D. Rädler, Jody Albright, Melissa VerHague, Erika T. Rezeli, Daniel Roth, John E. French, Charles M. Perou, Stephen D. Hursting, Michael F. Coleman

**Affiliations:** 1 Department of Nutrition, University of North Carolina, Chapel Hill, NC 27599, USA; 2 Department of Genetics, University of North Carolina, Chapel Hill, NC 27599, USA; 3 Nutrition Research Institute, University of North Carolina, Kannapolis, NC 28081, USA; 4 Lineberger Comprehensive Cancer Center, University of North Carolina, Chapel Hill, NC 27599, USA

**Keywords:** obesity, triple-negative breast cancer, model development, orthotopic transplantation

## Abstract

**Simple Summary:**

Transplanting cell lines into the mammary fat pad of lean and obese mice is a powerful tool to understand how breast cancer is promoted by obesity. However, for this approach to be effective, well-characterized and appropriate cell lines are needed. Here, we have developed four readily tumorigenic claudin-low triple-negative breast cancer cell lines from tumors arising from C3-TAg transgenic C57BL6 mice (B6TAg). We employ transcriptomic analysis of in vitro and in vivo samples to delineate distinct transcriptomic signatures in each cell line. We demonstrate that tumor progression of the three most distinct cell lines was accelerated by diet-induced obesity. Taken together, our data establish these B6TAg cell lines as potentially potent tools to delineate how obesity promotes triple-negative breast cancer progression.

**Abstract:**

Obesity is an established risk and progression factor for triple-negative breast cancer (TNBC), but preclinical studies to delineate the mechanisms underlying the obesity-TNBC link as well as strategies to break that link are constrained by the lack of tumor models syngeneic to obesity-prone mouse strains. C3(1)/SV40 T-antigen (C3-TAg) transgenic mice on an FVB genetic background develop tumors with molecular and pathologic features that closely resemble human TNBC, but FVB mice are resistant to diet-induced obesity (DIO). Herein, we sought to develop transplantable C3-TAg cell lines syngeneic to C57BL/6 mice, an inbred mouse strain that is sensitive to DIO. We backcrossed FVB-Tg(C3-1-TAg)cJeg/JegJ to C57BL/6 mice for ten generations, and spontaneous tumors from those mice were excised and used to generate four clonal cell lines (B6TAg1.02, B6TAg2.03, B6TAg2.10, and B6TAg2.51). We characterized the growth of the four cell lines in both lean and DIO C57BL/6J female mice and performed transcriptomic profiling. Each cell line was readily tumorigenic and had transcriptional profiles that clustered as claudin-low, yet markedly differed from each other in their rate of tumor progression and transcriptomic signatures for key metabolic, immune, and oncogenic signaling pathways. DIO accelerated tumor growth of orthotopically transplanted B6TAg1.02, B6TAg2.03, and B6TAg2.51 cells. Thus, the B6TAg cell lines described herein offer promising and diverse new models to augment the study of DIO-associated TNBC.

## 1. Introduction

Breast cancer (BC) is a heterogenous disease composed of several subtypes defined by molecular markers and immunohistochemical expression of hormone receptors. Triple-negative breast cancer (TNBC) lacks expression of estrogen receptor (ER), progesterone receptor (PR), and overexpression of human epidermal growth factor receptor 2 (HER2) [1,2,3]. TNBCs represent 15–20% of BC cases in the United States and predominantly exhibit claudin-low or basal-like transcriptomic profiles that are characteristically aggressive and difficult to treat [4,5]. Moreover, obesity is an established risk and progression factor for TNBC in epidemiologic and preclinical studies [6,7].

A powerful preclinical approach to interrogate how obesity promotes TNBC progression, as well as identify interventions to mitigate the pro-cancer effects of obesity, involves orthotopic transplantation of syngeneic TNBC cell lines into the mammary fat pad of mice [8]. This approach enables assessment of obesity-driven tumor progression absent confounding effects during tumor initiation, allows for genetic manipulation, and facilitates scheduling tumor generation at any timepoint following an intervention [8]. These models also have several practical advantages relative to genetically engineered or carcinogen-induced models, including lower vivarium costs due to use of standard, readily purchased immune-intact mouse strains, such as C57BL/6 mice, and relatively rapid tumor growth. Moreover, syngeneic models facilitate study of the tumor microenvironment, as the transplanted cells, extracellular matrix, and host factors such as T cells, macrophages and other immune and stromal cells all have the same genetic background [9]. To investigate the role of obesity in the context of TNBC progression, it is important to use appropriate and well-characterized cell lines that are syngeneic to an obesity-sensitive rodent strain and reliably form tumors when orthotopically transplanted.

Female FVB-Tg(C3-1-TAg)cJeg/JegJ mice bearing the C3(1)/SV40 large T-antigen (TAg) transgene spontaneously develop mammary tumors that recapitulate the histological development of basal-like TNBC in humans [10,11]. Further, these tumors arise following inactivation of p53 and Rb as is frequently seen in TNBC [10]. However, insensitivity of FVB mice to diet-induced obesity (DIO), and a short tumor latency period (tumors arise as early as 10–12 weeks of age), limit the utility of this model for obesity-related research [12,13,14,15]. Conversely, C57BL/6 mice are commonly used to study obesity, as they gain weight and mirror the systemic metabolic perturbations observed in human obesity (e.g., hyperglycemia, insulin resistance, dyslipidemia) when chronically fed a high-fat diet ad libitum [8,16,17]. We have previously established that orthotopically transplanted E0771 cells and several Wnt-driven cell lines in female C57BL/6 mice develop mammary tumors that are readily accelerated by obesity [18,19,20]. The goal of this study was to develop and characterize reproducible C3TAg orthotopic transplant models of TNBC on a C57BL/6 background to facilitate studies of obesity and TNBC progression in a human-relevant model of p53- and Rb-deficient TNBC.

## 2. Methods

### 2.1. Generation of B6-C3TAg Mice and Associated Mammary Tumor Cell Lines

All animal studies were conducted in accordance with a protocol approved by the Institutional Animal Care and Use Committee at the University of North Carolina at Chapel Hill. C57BL/6 C3(1)-TAg transgenic mice were generated by backcrossing female FVB-Tg(C3-1-TAg)cJeg/JegJ mice (provided by the UNC Preclinical Research Unit) with wildtype C57BL/6 male mice for 10 generations. Tumors were excised from the first two female mice in the 10th generation that developed a spontaneous mammary tumor. Dissected tumor sections (~3 mm) were placed in chilled L-15 media supplemented with 10% FBS, 2 mM glutamine, 1% penicillin/streptomycin, and 1% amphotericin B for transport, then dissociated into a single cell suspension using a Miltenyi mouse tumor dissociation kit (Miltenyi Biotech, Gaithersburg, MD, USA) according to the manufacturer’s instructions. Cell suspensions were cultured at a density of 3 × 10^4^ cells/cm^2^ in RPMI-1640 media supplemented with 10% FBS, 2 mM glutamine, 1% penicillin/streptomycin, and 1% amphotericin B for two weeks to establish pools of isolated tumor cells from C3(1)-TAg transgenic mice on a C57BL/6 background.

### 2.2. Clonal Cell Line Establishment

To establish clonal cell lines, C3(1)-TAg tumor cell pools were seeded at 3 cells/mL, of which 100 μL was seeded into each well of several 96-well plates to achieve a Poisson distribution whereby 30% of the wells had one cell and 70% of the wells had no cells. Wells containing only one cell were identified 18 h later by phase contrast microscopy, allowed to proliferate, and sequentially passaged. This resulted in four distinct and readily proliferative clonal cell lines, hereafter referred to as B6TAg1.02, B6TAg2.03, B6TAg2.10, and B6TAg2.51 cells. Cells 10–15 passages from isolation were used in all experiments.

### 2.3. Tumor Cell Transplant Study

To determine the tumorigenicity of the four newly derived B6TAg cell lines, 96 8-week-old C57BL/6J female mice were purchased from Jackson Laboratories (Bar Harbor, ME, USA). All mice were fed a low-fat diet (10 kcal% from fat, DJ2450 Research Diets) ad libitum, randomized (n = 8/group) to be orthotopically injected into the fourth mammary fat pad with either (i) 5 × 10^4^, (ii) 1.5 × 10^5^, or (iii) 5 × 10^5^ of B6TAg1.02, B6TAg2.03, B6TAg2.10 or B6TAg2.51 cells. Tumors were measured using digital calipers three times per week. All mice transplanted with 1.5 × 10^5^ cells were euthanized once any group reached an average diameter of 1.5 cm via CO_2_ inhalation followed by cervical dissociation, and tumors were excised and weighed on a digital scale to determine tumor mass. Tumors grew in mice that received 5 × 10^4^ or 5 × 10^5^ cells until they ulcerated or reached 1.5 cm in any direction, at which time the mice were euthanized and tumor excised, weighed, and flash frozen. Gross examination of lung and liver tissue for evidence of metastatic disease was performed, but none was detected.

### 2.4. Diet-Induced Obesity Study

C57BL/6J female mice (8–10-week-old, n = 120) purchased from Jackson Laboratories (Bar Harbor, USA) were randomized following a 1-week acclimation period to receive either the same low-fat diet as above or a high-fat diet (60 kcal% fat, D12492 Research Diets, n = 60) ad libitum for 36 weeks. Control and DIO mice were block randomized by body weight (n = 20/cell line) to receive an orthotopic injection of B6TAg1.02, B6TAg2.03 or B6TAg2.51 cells (2.5 × 10^5^ cells/mouse) into the fourth mammary fat pad. Mice continued their respective diets until one group reached the tumor endpoint, defined as tumor volumes measuring 1200 mm^3^ or tumors beginning to ulcerate, at which time the mice were euthanized and tumors excised, weighed and flash frozen as above.

### 2.5. Microarray Transcriptomic Profiling of In Vitro and Transplanted B6TAg Cell Lines

Total RNA was extracted from flash frozen tumors following B6TAg transplantation using TRIzol Reagent (Invitrogen, Carlsbad, CA, USA) and E.Z.N.A. Total RNA Kit I (Omega Biotech, Norcross, GA, USA). Total RNA was extracted from in vitro cell lines using E.Z.N.A. Total RNA Kit I (Omega Biotech, Norcross, GA, USA). RNA concentration and integrity was determined using an Agilent TapeStation (Agilent, Santa Clara, CA, USA).

RNA isolated from B6TAg cells in vitro and orthotopically transplanted was subjected to microarray analysis by the Functional Genomics Core at the University of North Carolina. Labeled sense-strand cDNA was synthesized and fragmented from total RNA using a GeneChip^TM^ WT PLUS Reagent Kit (Applied Biosystems, Carlsbad, CA, USA). Affymetrix GeneTitan Hybridization Wash and Stain Kit for WT Arrays were used to prepare a hybridization cocktail with the labeled cDNA. An Affymetrix GeneTitan Instrument (Applied Biosystems) performed the hybridization, washing, staining, and scanning of the Affymetrix Clariom^TM^ S Mouse Assay HT (Applied Biosystems). Quality control was performed using Transcriptome Analysis Console Software v 4.0 (Applied Biosystems). Principal component analysis (PCA) and unsupervised hierarchal clustering by Euclidean distance was performed on the 500 most variably expressed genes identified by ANOVA F statistic. To identify pathways and processes that were transcriptionally distinct between the B6TAg cell lines, gene set enrichment analysis (GSEA) (v4.0.3) was conducted on transcriptomic profiles using the Hallmark gene sets [21,22]. False discovery rate (FDR) q-value < 0.05 was considered significant. Digital cytometry was performed using CIBERSORTx [23].

### 2.6. RNAseq of Spontaneous and Transplanted B6TAg Tumors

RNA from spontaneous and orthotopically transplanted B6TAg frozen tumors was isolated using a RNeasy Mini Kit (Qiagen, Germantown, MA, USA) according to manufacturer’s instructions. mRNA quality was assessed with an Aligent Bioanalyzer and a library was created using total RNA and the Illumina TruSeq mRNA sample preparation kit. Paired-end (2 × 50 bp) reads were sequenced by the UNC High Throughput Sequencing Facility (HTSF) on an Illumina HiSeq 2000/2500 sequencer (Illumina, San Diego, CA, USA). Fastq files were aligned using the mouse mm10 reference genome and STAR aligner algorithm [24]. Resulting BAM files were sorted, indexed, and read counts determined using Salmon. A median centered, log2-transformed RNAseq data subset of 1723 intrinsic genes, incorporating B6TAg tumors into an established panel of diverse mammary tumor models and normal mammary gland tissue from publicly available records (GSE118164, GSE124821, and GSE223630) was generated as previously described [5]. Samples were hierarchically clustered using the centroid method on the expression of 1723 previously defined murine intrinsic genes [4] using Cluster3.0 (V3.0) [25]. Subclusters of genes that represent luminal, basal, claudin-low and proliferation gene sets were extracted with a node correlation of >0.65.

### 2.7. Statistical Analysis

Differences in tumor mass, tumor volume, and body weight were determined by a one-way ANOVA followed by Tukey’s post hoc test when comparing three or more groups or an unpaired student’s *t*-test when comparing two groups. Tumor growth over time was determined by a two-way ANOVA repeated measures followed by Šídák post hoc test. Survival analysis was conducted using the Gehan–Breslow–Wilcoxon test followed by FDR correction for multiple hypothesis testing. All data are presented as mean ± SD. Statistical analysis was conducted using GraphPad Prism software V10.2.3 (GraphPad Software Inc., San Diego, CA, USA). Results were considered statistically significant where *p* or FDRq < 0.05.

## 3. Results

### 3.1. Generation of B6-C3TAg Mice and Associated TNBC Cell Lines That Harbor Distinct Transcriptomic Profiles

**Figure 1:**
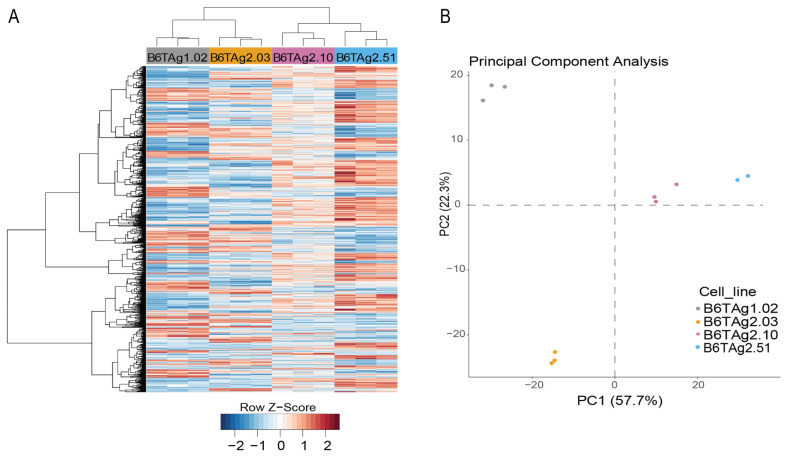
B6TAg cell lines are transcriptionally distinct models of breast cancer. (**A**) Unsupervised hierarchically clustered heat map and (**B**) principal component analysis (PCA) of the top 500 most variably expressed gene from B6TAg1.02, B6TAg2.03, B6TAg2.10, and B6TAg2.51 cell lines in vitro (n = 3).

B6-C3TAg mice developed mammary tumors at a mean age of 29 weeks, similar to prior observations of a mean latency of 32 weeks for development of TNBC in C57BL/6J mice bearing the C3TAg transgene [26]. Upon generating four new cell lines from tumors excised from B6-C3TAg mice (B6TAg1.02, B6TAg2.03, B6TAg2.10, B6TAg2.51), various transcriptomic analyses were performed. Using the 500 most variably expressed genes, hierarchal clustering and principal component analysis demonstrated that all four cell lines clustered separately from each other (Figure 1A,B). Thus, despite shared origins, B6TAg1.02, B6TAg2.03, B6TAg2.10, and B6TAg2.51 cell lines have unique gene expression patterns in vitro.

### 3.2. Immune Response and Growth/Survival-Related Signaling Transcriptomic Signatures Distinguish B6TAg Cells In Vitro

**Figure 2:**
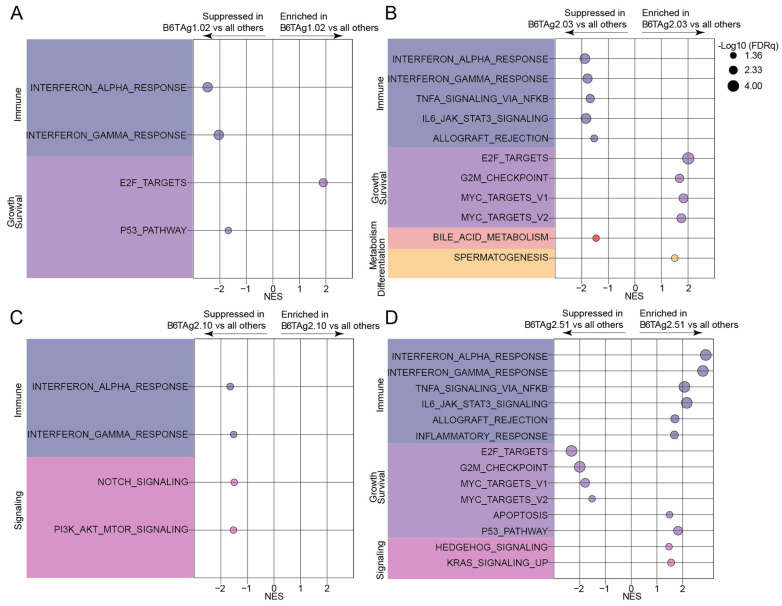
Immune- and growth and survival-related signatures define distinct transcriptomic profiles of B6TAg cell lines in vitro. Gene set enrichment analysis (GSEA) using Hallmark gene sets of (**A**) B6TAg1.02, (**B**) B6TAg2.03, (**C**) B6TAg2.10, and (**D**) B6TAg2.51, relative to all other cell lines. Statistical significance is defined as FDR q < 0.05.

To identify gene expression signatures differentially enriched across the B6TAg cell lines, we conducted GSEA [21] using the molecular signatures database (MSigDB) Hallmark gene sets [22]. Overall, in vitro gene expression profiles of B6TAg2.51 cells had the most significantly altered pathways compared with the other cell lines. B6TAg2.51 cells showed enrichment of immune-sensing-related pathways and suppression of MYC- and proliferation-related signatures. In contrast, B6TAg1.02 and B6TAg2.10 cells showed the lowest levels of immune-sensing enrichments, while B6TAg2.03 cells had the highest levels of MYC and E2F target enrichment relative to the other cell lines (Figure 2A–D, Supplementary Figure S1A–F). Specifically, marked enrichment of immune-sensing-related pathways such as interferon signaling, inflammatory response and allograft rejection was evident in B6TAg2.51 cells relative to all other cell lines (Figure 2D, Supplementary Figure S1A–C). Conversely, MYC signaling, E2F targets and G2M checkpoint gene sets were enriched in all other cell lines relative to B6TAg2.51 cells (Figure 2D, Supplementary Figure S1A–C). Relatively fewer gene sets were significantly different when comparing B6TAg1.02, B6TAg2.03, and B6TAg2.10 cells. Interferon-related signaling was enriched in both B6TAg2.03 and B6TAg2.10 cells relative to B6TAg1.02 cells (Figure 2A–C, Supplementary Figure S1D,E). Myeloid cells, particularly monocytes and macrophages, predominated the TME of tumors from all B6TAg cell lines, with approximately 25% lymphocytes present as determined by CIBERSORTx digital cytometry (Supplementary Figure S3A–D).

### 3.3. Orthotopically Transplanted B6TAg Cell Lines Reliably form Tumors in C57BL/6J Mice

**Figure 3:**
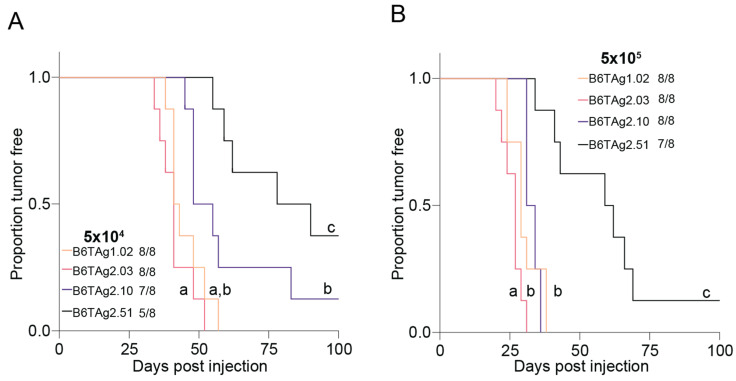
B6TAg cell lines are readily tumorigenic and progress at distinct rates. Survival analysis of mice orthotopically injected with (**A**) 5 × 10^4^ and (**B**) 5 × 10^5^ B6TAg1.02, B6TAg2.03, B6TAg2.10, B6TAg2.51 lines. Gehan–Breslow–Wilcoxon test followed by FDR correction. Different letters indicate significant differences between groups.

To determine if the B6TAg cell lines differentially form tumors in mice, we orthotopically injected 5 × 10^4^ cells and 5 × 10^5^ cells of each B6TAg cell line into the fourth mammary fat pad of lean female C57BL/6J mice. Overall, >90% of mice (59/64) developed tumors within 100 days (Figure 3A,B). When orthotopically transplanted at 5 × 10^4^ cells per mouse, B6TAg2.51 cells showed slower tumor progression than all other cell lines. Tumors from B6TAg2.10 cells progressed more quickly than B6TAg2.51 cells, but more slowly than B6TAg2.03 cells. B6TAg2.03 and B6TAg1.02 tumors progressed most quickly (Figure 3A). Injection of 5 × 10^5^ cells resulted in similar overall survival to that seen following transplantation of 5 × 10^4^ cells (Figure 3B). However, tumor free survival was limited by injection of 5 × 10^5^ cells relative to 5 × 10^4^ cells (Figure 3B).

**Figure 4:**
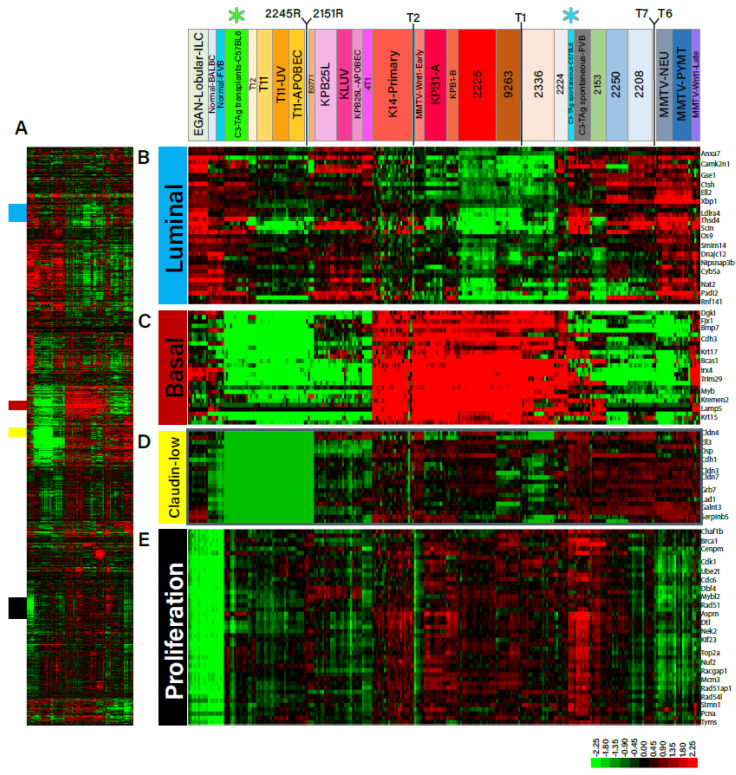
Orthotopic injection of B6TAg cell lines results in claudin-low breast cancer tumors. Unsupervised hierarchical clustering was performed on spontaneous (n = 3) (blue asterisk) and orthotopically injected (n = 12) C3TAg mammary tumors (green asterisk) with 241 reference samples from normal mammary and mouse breast cancer models using expression of 1723 intrinsic genes (upper colored bar). Spontaneous C3TAg tumors from C57BL/6 and FVB backgrounds cluster together as basal-like, while transplanted B6TAg tumors cluster with bona fide claudin-low T11 and T12 tumors. (**A**) Median centered gene expression of all intrinsic genes highlighting Luminal (blue), Basal (red), Claudin-low (yellow), and Proliferation (black) subclusters. Luminal (**B**), Basal (**C**), Claudin-low (**D**), and Proliferation (**E**) subclusters identified within intrinsic gene expression expanded to display relative expression of genes comprising the subcluster.

Next, we sought to determine whether spontaneous B6TAg tumors and those arising from orthotopic transplantation of B6TAg cell lines cluster with other models of basal-like breast cancer. The transcriptomic profile of spontaneous and orthotopically injected B6TAg tumors was determined by RNAseq followed by unsupervised hierarchical clustering with a library of other established models of murine breast cancer. Expression of 1723 intrinsic genes across 241 reference samples, 12 orthotopically transplanted, and 3 spontaneous B6TAg tumors was subject to hierarchical clustering. Spontaneous B6TAg tumors clustered closely with other basal-like models including spontaneous FVB/N C3TAg tumors. In contrast, our orthotopically transplanted B6TAg tumors were highly distinct and clustered separately from spontaneous B6TAg tumors due to prominent claudin-low features (Figure 4A–E).

### 3.4. Immune Response, Growth and Survival-Related Signaling, and Steroid Signaling Transcriptomic Signatures Distinguish B6TAg Tumors In Vivo

**Figure 5:**
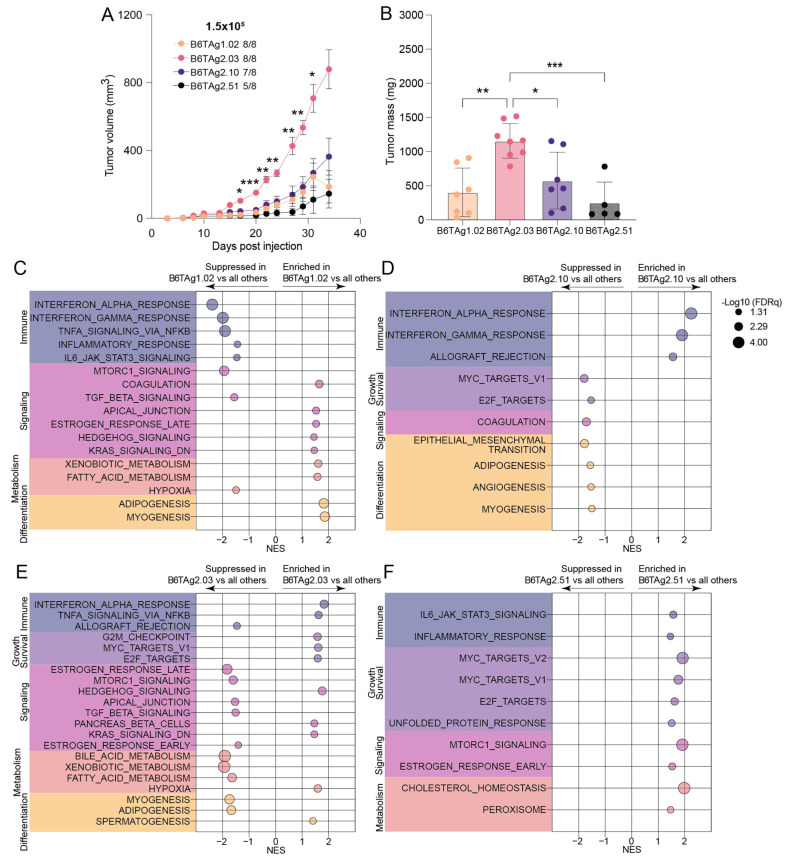
Orthotopic injection of B6TAg cell lines results in transcriptomic signatures of distinct tumor microenvironments. (**A**) Tumor volume and (**B**) tumor mass following orthotopic injection of 1.5 × 10^5^ B6TAg cells. Gene set enrichment analysis (GSEA) of Hallmark gene sets enriched in tumors generated by orthotopic injection of (**C**) B6TAg1.02, (**D**) B6TAg2.10, (**E**) B6TAg2.03, and (**F**) B6TAg2.51 relative to all other cell lines. Two-way ANOVA repeated measures followed by Šídák’s post hoc test (**A**). One-way ANOVA followed by Tukey post hoc test (**B**). (**D**–**F**) Statistical significance is defined as FDR q < 0.05 for GSEA. n = 5–8/group. Asterisks indicate: * *p* < 0.05, ** *p* < 0.01, *** *p* < 0.001.

To assess differences in biological pathways and processes, we isolated RNA from orthotopically transplanted tumors grown for 35 days in lean female C57BL/6J mice and conducted GSEA using the Hallmark gene sets. Tumors from B6TAg2.03 cells grew more rapidly and were larger at endpoint than all other groups (Figure 5A,B). B6TAg1.02 transplanted tumors had suppressed immune-related and enrichment of several signaling and metabolic signatures relative to tumors from all other cell lines (Figure 5C). B6TAg2.10 cells formed tumors enriched in several immune-related gene sets and had suppressed levels of growth and survival-, signaling- and differentiation-related pathways (Figure 5D). B6TAg2.03 transplanted tumors, relative to all other tumors, demonstrated enrichment of hypoxia and numerous growth and survival-related gene sets (Figure 5E). Relative to the other cell lines, B6TAg2.51 cells generated tumors enriched in immune- and growth and survival-related gene sets (Figure 5F). Overall rapid tumor growth correlated with strong enrichment of growth and survival related gene sets such as G2M checkpoint and E2F targets, and suppression of markers of antitumor immunity.

Immune-related gene sets were enriched in B6TAg2.03 tumors relative to B6TAg2.51 tumors (Supplementary Figure S2A), unlike in in vitro findings where B6TAg2.51 cells demonstrated the greatest enrichment of these gene sets. However, similar to in vitro findings, B6TAg2.51 tumors were characterized by an enrichment of immune-related gene sets relative to B6TAg1.02 tumors (Supplementary Figure S2B). B6TAg2.10 and B6TAg2.51 tumors had mixed enrichment of immune-related gene sets, with some associated with B6TAg2.10 and others associated with B6TAg2.51 (Supplementary Figure S2C). B6TAg1.02 tumors relative to tumors from all other cell lines demonstrated suppression of immune-related gene sets but enrichment in differentiation-related gene sets (Supplementary Figure S2B,D,E). B6TAg2.03 tumors showed suppressed steroid-related signaling relative to all other groups, and enrichment of G2M checkpoint and E2F target gene sets relative to all tumors except for B6TAg2.51 tumors (Supplementary Figure S2A,D,F).

### 3.5. Orthotopically Transplanted B6TAg Tumor Growth Is Accelerated by High-Fat Diet-Induced Obesity

**Figure 6:**
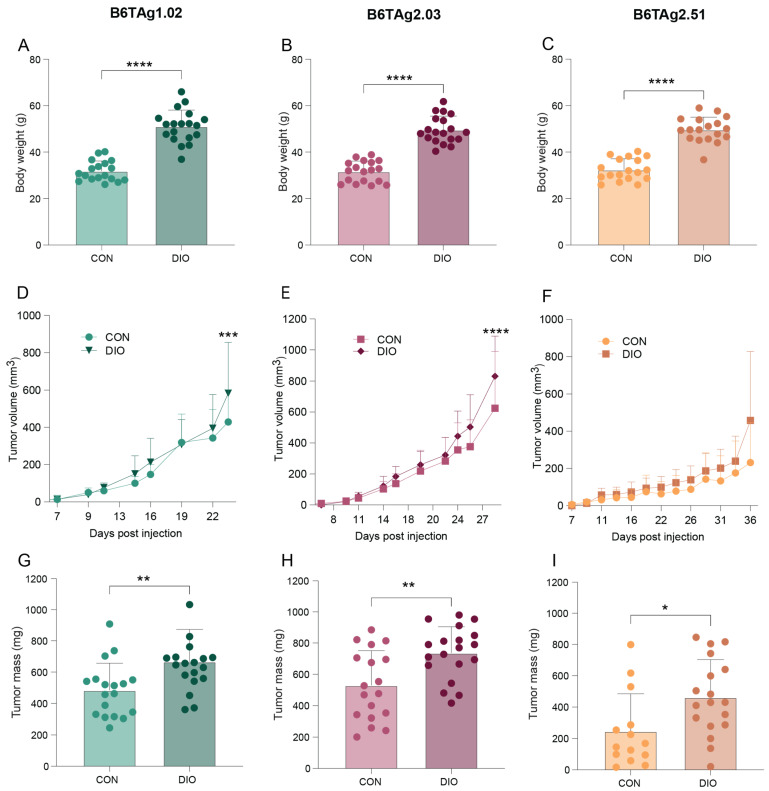
Obesity accelerates B6TAg tumor growth. (**A**–**C**) Terminal body weight, (**D**–**F**) Tumor growth over time, and (**G**–**I**) terminal tumor mass of control (CON) and DIO mice injected with B6TAg1.02, −2.03, and −2.51 cells. Data presented as mean ± SD. Unpaired students *t*-tests (**A**–**C**,**G**–**I**) or two-way ANOVA repeated measures followed by Šídák’s post hoc test (**D**–**F**). n = 14–19/group. Asterisks indicate: * *p* < 0.05, ** *p* < 0.01, *** *p* < 0.001, **** *p* < 0.0001.

To determine whether B6TAg tumors are sensitive to the protumor effects of obesity, we orthotopically transplanted B6TAg1.02, B6TAg2.03, and B6TAg2.51 cells into C57BL/6J female mice chronically fed a control or DIO diet. Owing to similarity between B6TAg2.10 and B6TAg1.02 cells in terms of transplanted tumor growth and transcriptional profiles, the B6TAg2.10 cell line was not selected for additional evaluation. The DIO mice weighed more than control mice at study termination (Figure 6A–C). Tumors grew faster and were larger at endpoint for all three B6TAg cell lines tested in DIO mice relative to controls (Figure 6D–I). As seen in Figure 3, tumor progression following orthotopic transplantation of B6TAg2.51 cells was slower than that of other cell lines (Figure 6D–F).

## 4. Discussion

**Figure 7:**
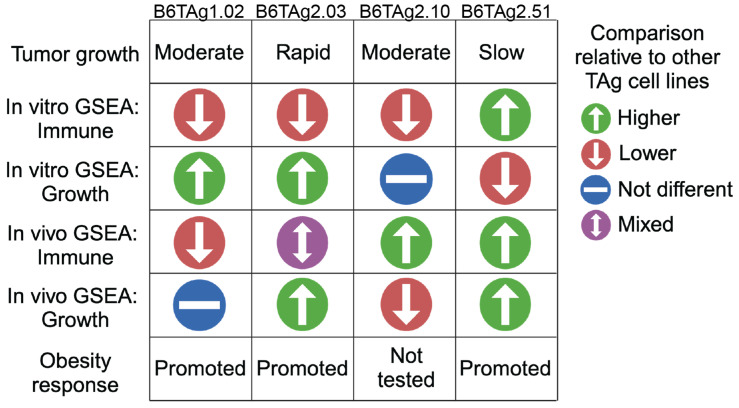
Summary of B6TAg cell line characteristics. Tumor progression, in vitro and in vivo GSEA results, and tumor progression in diet-induced obese mice are summarized.

We generated and characterized four novel transplantable claudin-low TNBC cell lines (i.e., B6TAg1.02, B6TAg2.03, B6TAg2.10, and B6TAg2.51) from C3(1)-TAg transgenic C57BL/6 mice. Each cell line induced tumors when orthotopically transplanted in C57BL/6 mice, with B6TAg2.51 tumors being the slowest growing (Figure 7). Differences in gene expression among the cell lines included: (i) immune-related gene sets were suppressed in B6TAg1.02 cells, and enriched in B6TAg2.51 cells, relative to the other cell lines, in vitro and in vivo (transplanted tumors); (ii) growth-related signature enrichment in vitro and in vivo, and hypoxia signature enrichment in vivo, in B6TAg2.03 cells, relative to the other cell lines; and (iii) several differentiation-related gene sets, including epithelial-to-mesenchymal transition, enriched in vivo in B6TAg2.10 cells, relative to the other cell lines (Figure 7). Importantly, DIO promoted tumor growth of orthotopically transplanted B6TAg1.02, B6TAg2.03, and B6TAg2.51 cells (Figure 7). Since previous transplantable C3TAg models were on an obesity-resistant FVB genetic background, these new syngeneic C3TAg orthotopic transplant models of TNBC on a C57BL/6 background represent an important new tool for mechanistic studies of obesity and TNBC progression.

While our work established transplantable cell lines that retain critical features of TNBC (e.g., claudin-low gene expression profile), prior work has shown that spontaneous TNBC tumors forming in C57BL/6 C3(1)-TAg mice grow more slowly than those in FVB C3(1)-TAg [26]. However, our work is the first to establish a panel of transplantable C3TAg cell lines that give rise to diverse tumor microenvironments with respect to both metabolic state and immune surveillance.

While various murine models of TNBC recapitulate essential features of human breast cancer, not all are able to appropriately address questions of obesity-driven cancers. For example, the metastatic 4T1 transplant model is a widely used murine model of TNBC [27,28]; however, this cell line was derived from BALB/c mice that are resistant to DIO [29]. In contrast, the E0771 orthotopic transplant model is derived from a spontaneous mammary tumor that developed in a C57BL/6 female mouse, and we and others have used this model to study obesity-driven TNBC progression [20,30,31,32]. Another mouse model of TNBC on a C57BL/6 background is the nonmetastatic “mesenchymal (M)-Wnt” cells and the metastatic “metM-Wnt^lung^ cells”, derived in our lab from a spontaneous mammary tumor excised from an MMTV-Wnt-1 transgenic mouse [18,19]. While orthotopically transplanted E0771 and Wnt-driven mammary tumors are readily responsive to systemic energy balance, they are driven by less common oncogenic drivers of human TNBC than p53 and Rb, and thus the B6TAg lines described here fill a gap in the field [33]. Like any orthotopic transplant model, a limitation of the new models described herein is that they utilize isolated cells that are fully transformed and readily tumorigenic, and thus cannot be used to address the role of oncogenic transformation or progression of preneoplastic lesions in obesity-driven TNBC.

## 5. Conclusions

Taken together, our findings suggest that the B6TAg transplantable tumors described herein: (i) reliably form TNBC tumors; (ii) generate tumor microenvironments with distinct metabolic and immune landscapes; (iii) are sensitive to modulation by DIO; (iv) provide a promising new resource for the study of obesity-associated TNBC; and (v) may have utility for testing new chemoprevention, chemotherapy, or immunotherapy regimens for TNBC in immune-intact orthotopic transplant models.

## Data Availability

The transcriptomic data associated with this paper are available through GEO at GSE272327 and GSE 272328. All other data are available from the corresponding author on request.

## Supplementary Materials

The following supporting information can be downloaded at: https://www.mdpi.com/article/10.3390/cancers16162803/s1, Supplementary Figure S1. Pairwise comparison of B6TAg cell lines reveals unique in vitro transcriptional signatures. Gene set enrichment analysis (GSEA) of Hallmark gene sets enriched in (A) B6TAg2.51 vs. B6TAg2.03, (B) B6TAg2.51 vs. B6TAg1.02, (C) B6TAg2.51 vs. B6TAg2.10, (D) B6TAg1.02 vs. B6TAg2.03, (E) B6TAg1.02 vs. B6TAg2.10, and (F) B6TAg2.03 vs. B6TAg2.10 cell lines. Statistically significance defined as FDR q < 0.05. Supplementary Figure S2. Pairwise comparison of B6TAg cell lines reveals unique in vivo transcriptional signatures. Gene set enrichment analysis (GSEA) of Hallmark gene sets enriched in (A) B6TAg2.51 vs. B6TAg2.03, (B) B6TAg2.51 vs. B6TAg1.02, (C) B6TAg2.51 vs. B6TAg2.10, (D) B6TAg1.02 vs. B6TAg2.03, (E) B6TAg1.02 vs. B6TAg2.10, and (F) B6TAg2.03 vs. B6TAg2.10 tumors. Statistically significance defined as FDR q < 0.05. Supplementary Figure S3. Digital cytometry of tumors following orthotopically transplanted B6TAg cell lines. CIBERSORTx digital cytometry was performed on transcriptomic profiles of tumors generated by orthotopic injection of B6TAg1.02 (A), B6TAg2.03 (B), B6TAg2.10 (C), and B6TAg2.51 (D).

## References

[1] Orrantia-Borunda E., Anchondo-Nunez P., Acuna-Aguilar L.E., Gomez-Valles F.O., Ramirez-Valdespino C.A., Mayrovitz H.N. (2022). Subtypes of Breast Cancer. Breast Cancer.

[2] Siegel R.L., Giaquinto A.N., Jemal A. (2024). Cancer statistics, 2024. CA A Cancer J. Clin..

[3] Gradishar W.J., Moran M.S., Abraham J., Abramson V., Aft R., Agnese D., Allison K.H., Anderson B., Burstein H.J., Chew H. (2023). NCCN Guidelines^®^ Insights: Breast Cancer, Version 4.2023. J. Natl. Compr. Cancer Netw..

[4] Pfefferle A.D., Herschkowitz J.I., Usary J., Harrell J.C., Spike B.T., Adams J.R., Torres-Arzayus M.I., Brown M., Egan S.E., Wahl G.M. (2013). Transcriptomic classification of genetically engineered mouse models of breast cancer identifies human subtype counterparts. Genome Biol..

[5] Rädler P.D., Wehde B.L., Triplett A.A., Shrestha H., Shepherd J.H., Pfefferle A.D., Rui H., Cardiff R.D., Perou C.M., Wagner K.-U. (2021). Highly metastatic claudin-low mammary cancers can originate from luminal epithelial cells. Nat. Commun..

[6] Berger E.R., Iyengar N.M. (2021). Obesity and Energy Balance Considerations in Triple-Negative Breast Cancer. Cancer J..

[7] Bohm M.S., Sipe L.M., Pye M.E., Davis M.J., Pierre J.F., Makowski L. (2022). The role of obesity and bariatric surgery-induced weight loss in breast cancer. Cancer Metastasis Rev..

[8] Glenny E.M., Coleman M.F., Giles E.D., Wellberg E.A., Hursting S.D. (2021). Designing Relevant Preclinical Rodent Models for Studying Links Between Nutrition, Obesity, Metabolism, and Cancer. Annu. Rev. Nutr..

[9] Stribbling S.M., Beach C., Ryan A.J. (2024). Orthotopic and metastatic tumour models in preclinical cancer research. Pharmacol. Ther..

[10] Green J.E., Shibata M.A., Yoshidome K., Liu M.L., Jorcyk C., Anver M.R., Wigginton J., Wiltrout R., Shibata E., Kaczmarczyk S. (2000). The C3(1)/SV40 T-antigen transgenic mouse model of mammary cancer: Ductal epithelial cell targeting with multistage progression to carcinoma. Oncogene.

[11] Herschkowitz J.I., Simin K., Weigman V.J., Mikaelian I., Usary J., Hu Z., Rasmussen K.E., Jones L.P., Assefnia S., Chandrasekharan S. (2007). Identification of conserved gene expression features between murine mammary carcinoma models and human breast tumors. Genome Biol..

[12] Kim D.H., Gutierrez-Aguilar R., Kim H.J., Woods S.C., Seeley R.J. (2013). Increased adipose tissue hypoxia and capacity for angiogenesis and inflammation in young diet-sensitive C57 mice compared with diet-resistant FVB mice. Int. J. Obes..

[13] Yoshidome K., Shibata M.A., Maroulakou I.G., Liu M.L., Jorcyk C.L., Gold L.G., Welch V.N., Green J.E. (1998). Genetic alterations in the development of mammary and prostate cancer in the C3(1)/Tag transgenic mouse model. Int. J. Oncol..

[14] Hu C.C., Qing K., Chen Y. (2004). Diet-induced changes in stearoyl-CoA desaturase 1 expression in obesity-prone and -resistant mice. Obes. Res..

[15] Devlin M.J., Robbins A., Cosman M.N., Moursi C.A., Cloutier A.M., Louis L., Van Vliet M., Conlon C., Bouxsein M.L. (2018). Differential effects of high fat diet and diet-induced obesity on skeletal acquisition in female C57BL/6J vs. FVB/NJ Mice. Bone Rep..

[16] Montgomery M.K., Hallahan N.L., Brown S.H., Liu M., Mitchell T.W., Cooney G.J., Turner N. (2013). Mouse strain-dependent variation in obesity and glucose homeostasis in response to high-fat feeding. Diabetologia.

[17] Surwit R.S., Feinglos M.N., Rodin J., Sutherland A., Petro A.E., Opara E.C., Kuhn C.M., Rebuffe-Scrive M. (1995). Differential effects of fat and sucrose on the development of obesity and diabetes in C57BL/6J and A/J mice. Metabolism.

[18] Dunlap S.M., Chiao L.J., Nogueira L., Usary J., Perou C.M., Varticovski L., Hursting S.D. (2012). Dietary energy balance modulates epithelial-to-mesenchymal transition and tumor progression in murine claudin-low and basal-like mammary tumor models. Cancer Prev. Res..

[19] O’Flanagan C.H., Rossi E.L., McDonell S.B., Chen X., Tsai Y.H., Parker J.S., Usary J., Perou C.M., Hursting S.D. (2017). Metabolic reprogramming underlies metastatic potential in an obesity-responsive murine model of metastatic triple negative breast cancer. NPJ Breast Cancer.

[20] Bowers L.W., Doerstling S.S., Shamsunder M.G., Lineberger C.G., Rossi E.L., Montgomery S.A., Coleman M.F., Gong W., Parker J.S., Howell A. (2022). Reversing the Genomic, Epigenetic, and Triple-Negative Breast Cancer–Enhancing Effects of Obesity. Cancer Prev. Res..

[21] Subramanian A., Tamayo P., Mootha V.K., Mukherjee S., Ebert B.L., Gillette M.A., Paulovich A., Pomeroy S.L., Golub T.R., Lander E.S. (2005). Gene set enrichment analysis: A knowledge-based approach for interpreting genome-wide expression profiles. Proc. Natl. Acad. Sci. USA.

[22] Liberzon A., Birger C., Thorvaldsdottir H., Ghandi M., Mesirov J.P., Tamayo P. (2015). The Molecular Signatures Database (MSigDB) hallmark gene set collection. Cell Syst..

[23] Newman A.M., Steen C.B., Liu C.L., Gentles A.J., Chaudhuri A.A., Scherer F., Khodadoust M.S., Esfahani M.S., Luca B.A., Steiner D. (2019). Determining cell type abundance and expression from bulk tissues with digital cytometry. Nat. Biotechnol..

[24] Dobin A., Davis C.A., Schlesinger F., Drenkow J., Zaleski C., Jha S., Batut P., Chaisson M., Gingeras T.R. (2013). STAR: Ultrafast universal RNA-seq aligner. Bioinformatics.

[25] de Hoon M.J.L., Imoto S., Nolan J., Miyano S. (2004). Open source clustering software. Bioinformatics.

[26] Sena I.F.G., Rocha B.G.S., Picoli C.C., Santos G.S.P., Costa A.C., Goncalves B.O.P., Garcia A.P.V., Soltani-Asl M., Coimbra-Campos L.M.C., Silva W.N. (2021). C(3)1-TAg in C57BL/6 J background as a model to study mammary tumor development. Histochem. Cell Biol..

[27] Turbitt W.J., Xu Y., Sosnoski D.M., Collins S.D., Meng H., Mastro A.M., Rogers C.J. (2019). Physical Activity Plus Energy Restriction Prevents 4T1.2 Mammary Tumor Progression, MDSC Accumulation, and an Immunosuppressive Tumor Microenvironment. Cancer Prev. Res..

[28] Shinde A., Wilmanski T., Chen H., Teegarden D., Wendt M.K. (2018). Pyruvate carboxylase supports the pulmonary tropism of metastatic breast cancer. Breast Cancer Res..

[29] Kaur P., Nagaraja G.M., Zheng H., Gizachew D., Galukande M., Krishnan S., Asea A. (2012). A mouse model for triple-negative breast cancer tumor-initiating cells (TNBC-TICs) exhibits similar aggressive phenotype to the human disease. BMC Cancer.

[30] Sugiura K., Stock C.C. (1952). Studies in a tumor spectrum. II. The effect of 2,4,6-triethylenimino-s-triazine on the growth of a variety of mouse and rat tumors. Cancer.

[31] Sipe L.M., Chaib M., Korba E.B., Jo H., Lovely M.C., Counts B.R., Tanveer U., Holt J.R., Clements J.C., John N.A. (2022). Response to immune checkpoint blockade improved in pre-clinical model of breast cancer after bariatric surgery. eLife.

[32] Ringel A.E., Drijvers J.M., Baker G.J., Catozzi A., García-Cañaveras J.C., Gassaway B.M., Miller B.C., Juneja V.R., Nguyen T.H., Joshi S. (2020). Obesity Shapes Metabolism in the Tumor Microenvironment to Suppress Anti-Tumor Immunity. Cell.

[33] Du X., Li G., Liu J., Zhang C., Liu Q., Wang H., Chen T. (2021). Comprehensive analysis of the cancer driver genes in breast cancer demonstrates their roles in cancer prognosis and tumor microenvironment. World J. Surg. Oncol..

