# Supplementary material for: Development and Characterization of Syngeneic Orthotopic Transplant Models of Obesity-Responsive Triple-Negative Breast Cancer in C57BL/6J Mice
Source: Cancers (Basel). 2024 Aug 9;16(16):2803. doi: 10.3390/cancers16162803 (PMC11352691; doi:10.3390/cancers16162803)
Supplement: Supplementary file 1 [file cancers-16-02803-s001.zip › C3TAg_Supp_FigureS3_V1.pdf]

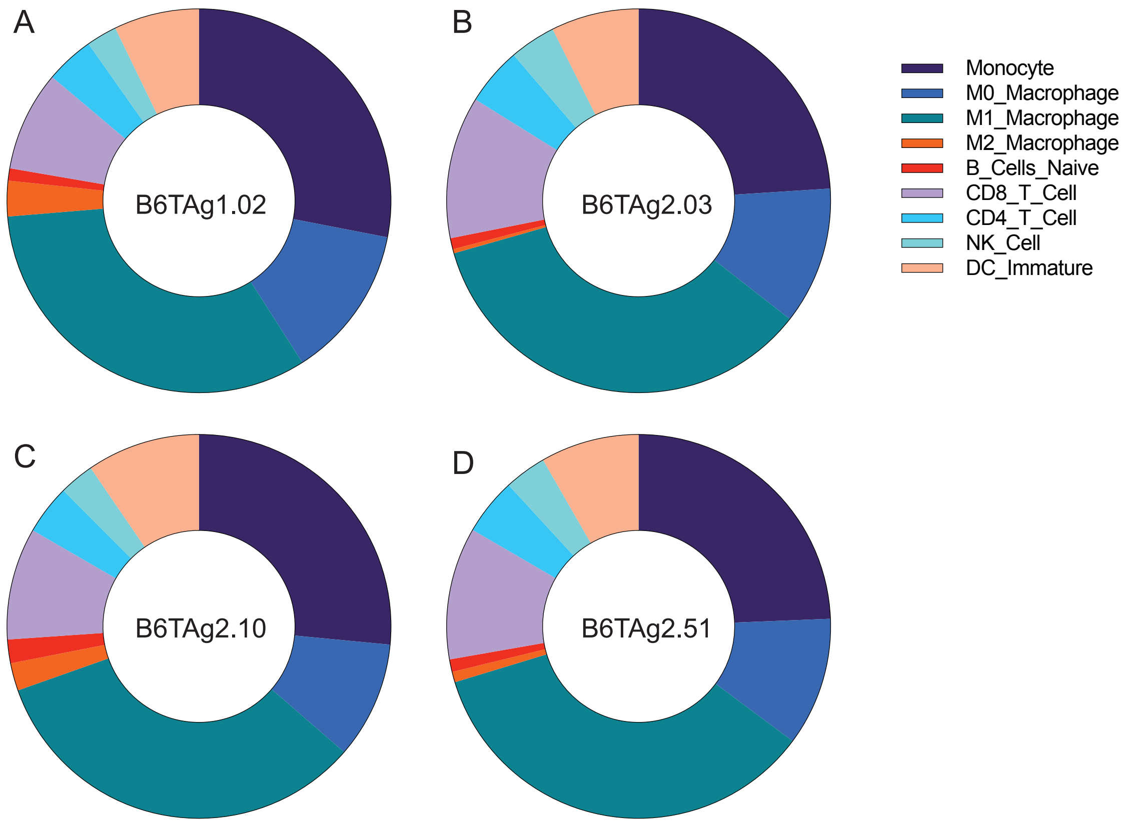
A
B
C
D
B6TAg1.02
B6TAg2.03
B6TAg2.10
B6TAg2.51
Monocyte
M0_Macrophage
M1_Macrophage
M2_Macrophage
B_Cells_Naive
CD8_T_Cell
CD4_T_Cell
NK_Cell
DC_Immature
