# Supplementary figures and images for: Development and Characterization of Syngeneic Orthotopic Transplant Models of Obesity-Responsive Triple-Negative Breast Cancer in C57BL/6J Mice
Source: Cancers (Basel). 2024 Aug 9;16(16):2803. doi: 10.3390/cancers16162803 (PMC11352691; doi:10.3390/cancers16162803)

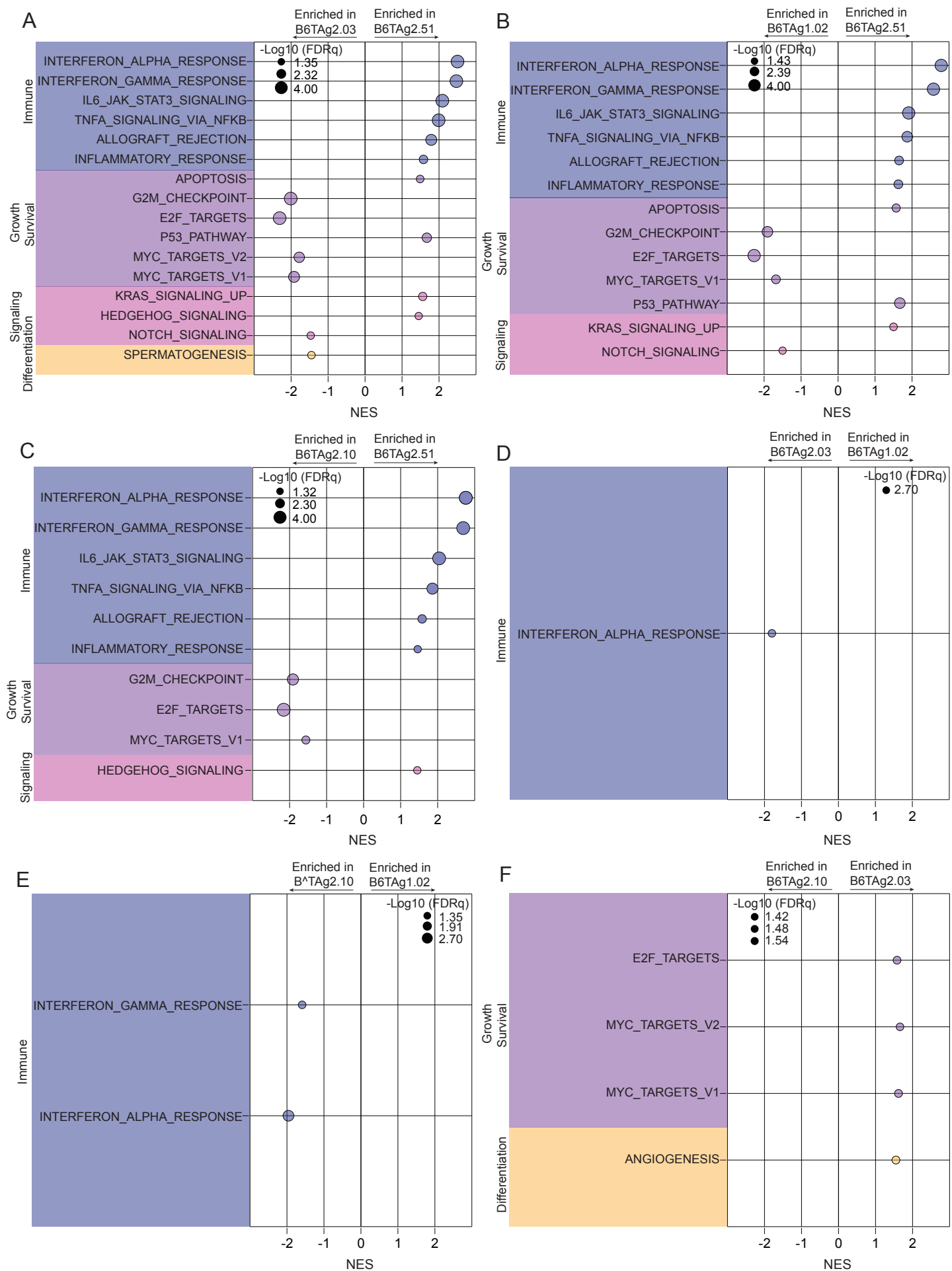

Supplement: Supplementary file 1 [file cancers-16-02803-s001.zip › C3TAg_Supp_FigureS1_V4.pdf]

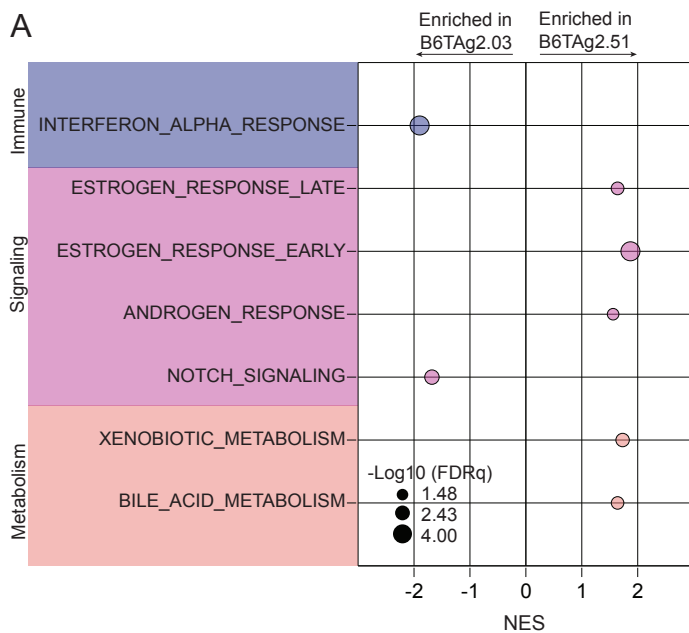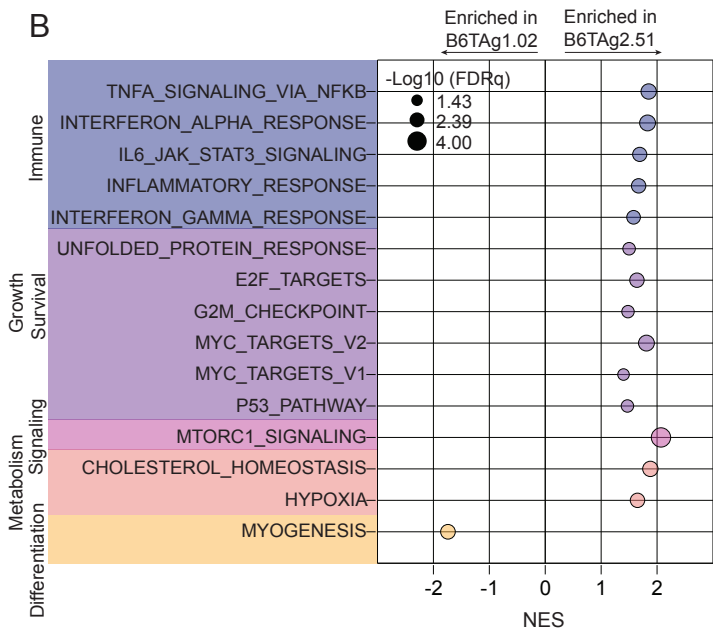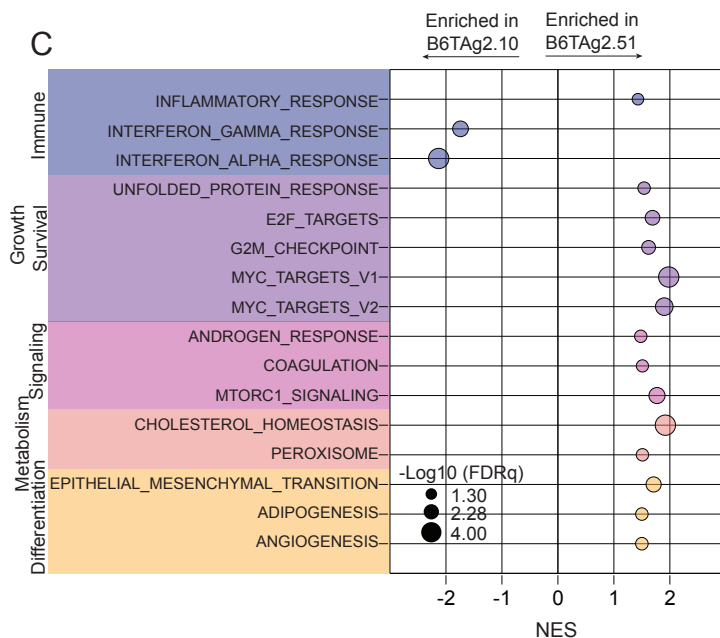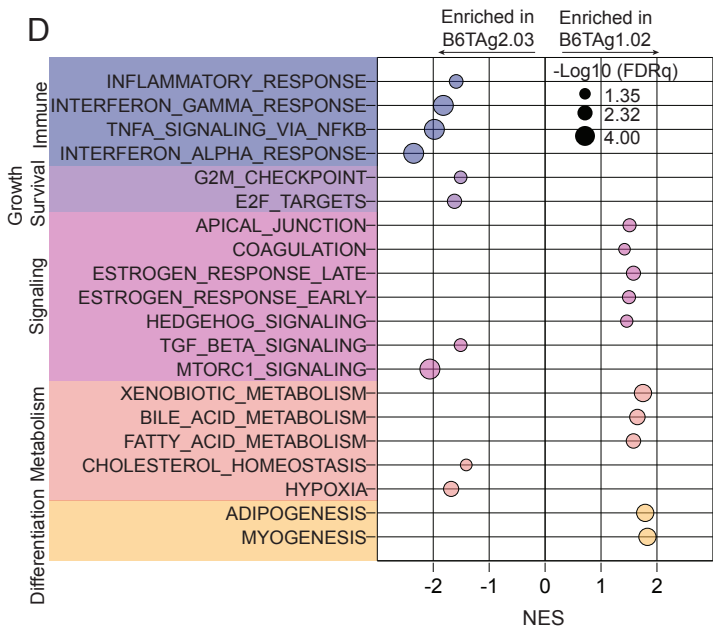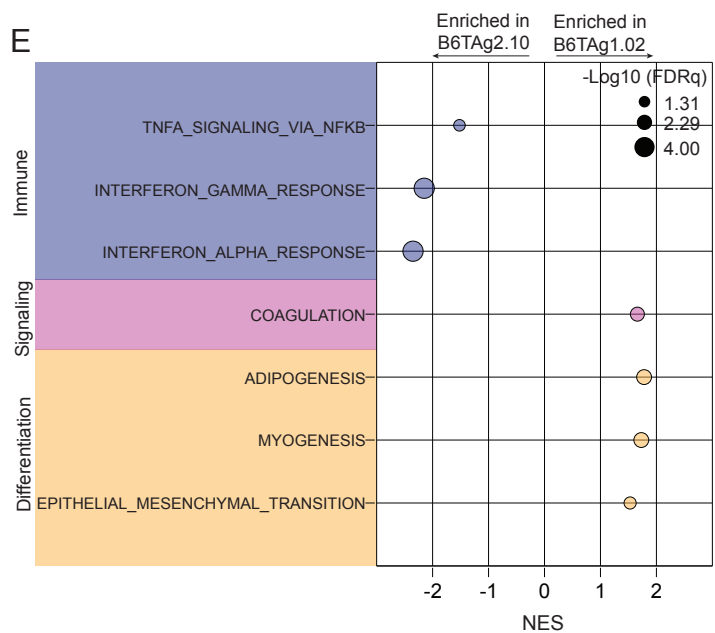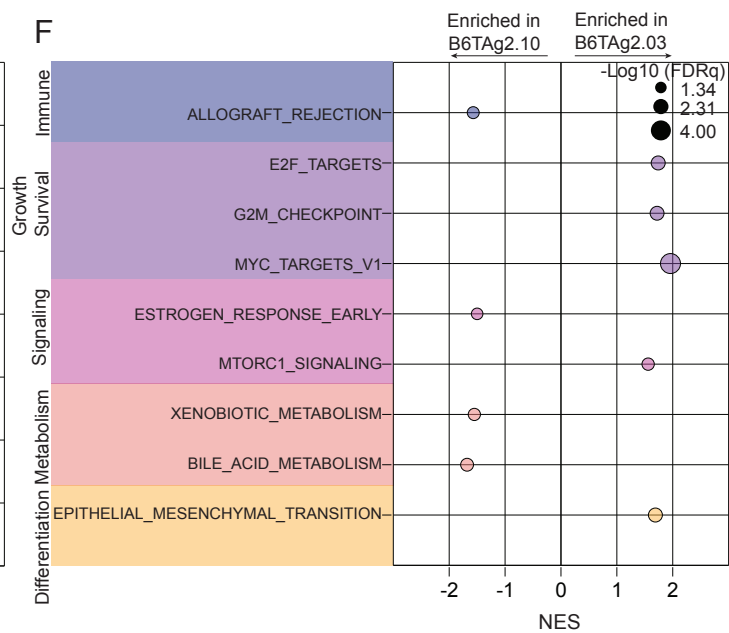

Supplement: Supplementary file 1 [file cancers-16-02803-s001.zip › C3TAg_Supp_FigureS2_V4.pdf]
